# Methyl-Donor Micronutrient for Gestating Sows: Effects on Gut Microbiota and Metabolome in Offspring Piglets

**DOI:** 10.3389/fnut.2021.675640

**Published:** 2021-06-07

**Authors:** Qin He, Tiande Zou, Jun Chen, Jia He, Li Jian, Fei Xie, Jinming You, Zirui Wang

**Affiliations:** ^1^Key Laboratory of Animal Nutrition in Jiangxi Province, Jiangxi Agricultural University, Nanchang, China; ^2^Key Innovation Center for Industry-Education Integration of High-Quality and Safety Livestock Production in Jiangxi Province, Jiangxi Agricultural University, Nanchang, China

**Keywords:** gilts, metabolic profiles, methyl-donor micronutrient, microbial community, offspring piglets

## Abstract

This study aimed to investigate the effects of maternal methyl-donor micronutrient supplementation during gestation on gut microbiota and the fecal metabolic profile in offspring piglets. Forty-three Duroc × Erhualian gilts were assigned to two dietary groups during gestation: control diet (CON) and CON diet supplemented with MET (folic acid, methionine, choline, vitamin B6, and vitamin B12). The body weights of offspring piglets were recorded at birth and weaning. Besides this, fresh fecal samples of offspring piglets were collected at 7, 14, and 21 days. The gut microbiota composition, metabolic profile, and short-chain fatty acid (SCFA) profiles in the fecal samples were determined using 16S rDNA sequencing, liquid chromatography-mass spectrometry metabolomics, and gas chromatography methods, respectively. The results showed that maternal methyl-donor micronutrient supplementation increased the microbiota diversity and uniformity in feces of offspring piglets as indicated by increased Shannon and Simpson indices at 7 days, and greater Simpson, ACE, Chao1 and observed species indices at 21 days. Specifically, at the phylum level, the relative abundance of Firmicutes and the Firmicutes to Bacteroidetes ratio were elevated by maternal treatment. At the genus level, the relative abundance of SCFA-producing *Dialister, Megasphaera*, and *Turicibacter*, and lactate-producing *Sharpea* as well as *Akkermansia, Weissella*, and *Pediococcus* were increased in the MET group. The metabolic analyses show that maternal methyl-donor micronutrient addition increased the concentrations of individual and total SCFAs of 21-day piglets and increased metabolism mainly involving amino acids, pyrimidine, and purine biosynthesis. Collectively, maternal methyl-donor micronutrient addition altered gut microbiota and the fecal metabolic profile, resulting in an improved weaning weight of offspring piglets.

## Introduction

The gut microbiota and fecal metabolic profile of neonates are closely related to immunity, disease, growth, and development. The colonization of microbial communities in the intestines is a key process in infant development (1); the intestinal microbial establishment is mainly determined and easily affected by diet, mother-to-child transmission, and the environment (2). The indigestible nutrients that are metabolized by bacteria in the neonatal intestines improve energy harvesting for growth but expose developing mammals to a variety of chemicals (3). The stability of gut microbiota systems can adapt to such environmental fluctuations. Therefore, a dysbiosis in the gut microbial community not only results in higher disease risk, but also causes short- and long-lasting adverse effects on neonates' health. Diet is an important determinant of maternal offspring's microbial communities and health outcomes. Maternal nutritional status has a great influence on fetal development, pregnancy outcome, and offspring disease development (4–6). Therefore, a specific diet plan for pregnant women may be a cost-effective intervention to promote intestinal microbiota colonization of offspring.

Methyl-donor micronutrients (MET), including folate, choline, betaine, methionine, cobalamin, pyridoxine, and so forth, are demonstrated to participate in the synthesis of nucleotides, proteins, and lipids via epigenetic mechanisms (7); meanwhile, the epigenetic mechanisms also modify the metabolome (8). However, the intergenerational effect of MET on the physiological metabolism of offspring in the early stage is hardly documented. Maternal diet during pregnancy may have a significant impact on the establishment of the neonatal microbiota, and may play a role in infant development (9). Additionally, there is increasing evidence that microbiota-derived metabolites are key factors regulating animal metabolism, growth, and development (10). Maternal-MET supplementation induces a specific intestinal microenvironment, limiting pathobiont colonization [such as adherent-invasive Escherichia coli (AIEC)] of the offspring gut (11). Conversely, other studies report that maternal-MET supplementation or deficiency leads to an increase in the sensitivity to colitis in the offspring (12, 13), suggesting the central role of MET in the intestinal microenvironment. Therefore, differences in the metabolome of control and MET offspring in the early stages after birth need to be further studied.

In our previous study, we found no differences in the reproduction performance of sows between CON and MET (number of total born, born alive, and weaned piglets per litter, etc.). However, maternal MET supplementation during pregnancy promotes skeletal muscle differentiation and maturity in newborn and weaning pigs and improves the growth performance of the offspring (14). This means that some changes in the suckling piglets may be important for growth and development. Therefore, this study was conducted to test the hypothesis that maternal methyl-donor micronutrient supplementation during gestation could beneficially regulate the gut microbiota and fecal metabolic profile and enhance the growth performance of offspring piglets.

## Materials and Methods

### Ethics Statement

This study was conducted under the Chinese guidelines for animal welfare. All animal experiments were approved by the Animal Care and Use Committee of Jiangxi Agricultural University (Ethic Approval Code: JXAUA01).

### Animals and Experimental Design

A total of 43 crossbred gilts (Duroc × Erhualian, body weight: 102.8 ± 6.3 kg) were artificially inseminated and then allotted by body weight to two dietary treatments. The two experimental diets were supplemented with or without methyl-donor micronutrients in the basal diet. There were 21 and 22 gilts in the control group (CON group) and Methyl-donor micronutrients group (MET group), respectively. The MET-supplemented diet contained 4,700 mg kg^−1^ methionine (CJ BIO, Malaysia, purity ≥99%), 16.3 mg kg^−1^ folic acid (Sigma-Aldrich, St. Louis, MO, USA, purity ≥97%), 2,230 mg kg^−1^ choline (NB GROUP, Shangdong, China, purity = 60%), 0.15 mg kg^−1^ vitamin B12 (Sigma-Aldrich, St. Louis, MO, USA, purity ≥98%), and 1,180 mg kg^−1^ vitamin B6 (Jiangxi Tianxin Pharmaceutical, Jiangxi, China, purity ≥98%). The dosage of methyl-donor micronutrients was added according to previous studies (15–18). Dietary treatment started at the last insemination and lasted until parturition. Ingredients and composition of pregnant gilt diets are show in Supplementary Table 1. During gestation, sows were fed 2.28 kg/day during days 1–80, 2.40 kg/day during days 80–90, and 3.00 kg/day of diet from day 91 until farrowing. Sows were fed discretely twice daily at 0800 and 1,400 h with 50% of the daily ration during each feeding. On day 110 of gestation, sows were transferred to farrowing pens. After parturition, all sows received a standard lactation diet (Supplementary Table 2) three times per day (i.e., 0800, 1,200, and 1,500 h). Piglets were weaned at 24 days. All animals were free to drink water. The experiment began with 54 gilts, 27 gilts per treatment. Pregnancy was confirmed by ultrasonic examination 30 days post-mating; 11 gilts were eliminated due to failure of pregnancy.

### Data and Sample Collection

#### Sample Collection

After parturition, body weights (BW) were measured at birth and weaning (24 days). Six litters per group were selected, and one median-birth-weight piglet from each litter were sampled for feces collection at days 7, 14, and 21 and then snap-frozen in liquid nitrogen and stored at −80°C for gut microbiota, short-chain fatty acid (SCFA), and metabolomics analyses. Fecal 16S rDNA sequencing and liquid chromatography-mass spectrometry (LC-MS) metabolomics were performed according to the manufacturer's instructions (Shanghai Applied Protein Technology, Shanghai, China).

#### Serum S-Adenosylmethionine and Homocysteine

Serum from newborn and weaning offspring was analyzed for S-adenosylmethionine (SAM) and homocysteine (Hcy) using the enzyme-linked immunosorbent assay (ELISA) kits purchased from MLBIO (Shanghai, China).

#### Fecal DNA Extraction and 16S rDNA Gene Amplicon Sequencing Analysis

The total genomic DNA was extracted from fecal samples with the Cetyltrimethylammonium Bromide (CTAB) method. DNA concentration and purity were monitored on an agarose gel. The DNA was then diluted accordingly using sterile water. The bacterial 16S rDNA gene of various regions (3–4) was amplified by polymerase chain reaction (PCR) using a specific primer (Uni340F: 5′-CCTAYGGGRBGCASCAG-3′, Uni806R: 5′-GGACTACNNGGGTATCTAAT-3′). The PCR product was detected with 2% agarose gel and purified with the Qiagen Gel Extraction Kit (Qiagen, Germany) following the manufacturer's instructions. The library was generated by TruSeq^®^ DNA PCR-Free Sample Preparation Kit (Illumina, USA) and quantified using a Qubit @ 2.0 Fluorometer (Thermo Scientific). Finally, 250 base pair paired-end sequencing was performed using the Illumina HiSeq 2500 platform.

Sequencing data were analyzed using the quantitative insights into microbial ecology (QIIME) (V1.9.1, http://qiime.org/scripts/split_libraries_fastq.html). Paired-end reads were merged using Fast Length Adjustment of Short reads (FLASH, V1.2.7, http://ccb.jhu.edu/software/FLASH/). The clean data were obtained by specific splice and filtering of the raw sequence data. Sequences with ≥97% similarity were assigned to the same operational taxonomic units (OTUs) and the OTU was screened for further annotation; analysis was performed using Uparse software (Uparse v7.0.1001, http://drive5.com/uparse/). The abundance information of OTUs was normalized using the serial number standard corresponding to the sample with the least sequence and both alpha (observed species, Chao1, Shannon, Simpson, ACE) and beta diversity (principal coordinate analysis (PCoA) and unweighted pair-group method with arithmetic means) were performed based on the normalized data of OTU abundance information. The alpha and beta diversity were calculated with QIIME and displayed with R software (Version 2.15.3). PCoA was based on unweighted UniFrac distances using the *WGCNA, stat*, and *ggplot2* packages in R. To determine the significance test of community structure differences between groups, permutational multivariate analysis of variance (PERMANOVA, Adonis procedure with 999 permutations) was performed in R to calculate *P*-values. Additionally, to further explore the differences in the community structure (phylum and genus) between the two groups of samples, the linear discriminant analysis (LDA) effect size (LEfSe) method was used to compare the differences in the taxonomic levels; the LDA score was set at 2.0 for a biomarker. In LEfSe analysis, the non-parametric factorial Kruskal–Wallis (KW) sum-rank test was used to detect all species with significant differential abundance, and the Wilcoxon rank-sum test was used to investigate biological consistency among subclasses.

#### Concentration of SCFA in the Fecal

Approximately 1 g of fecal samples were weighed, diluted with 2 mL of ultra-pure water and centrifuged at 12,000 g (4°C) for 15 min to obtain a supernatant. Then 25% of metaphosphoric acid solution was added in a ratio of 9:1 before centrifugation at 3,000 g for 10 min. The supernatant was aspirated with a syringe and filtered through a 0.45 mm filter membrane. Acetic acid, propionic acid, isobutyric acid, butyric acid, isovaleric acid, valeric acid, and total SCFA were determined using capillary column gas chromatography (Shimadzu Gas Chromatography 2014, Japan; capillary column length was 30 m, inner diameter was 0.25 mm, and film thickness was 0.25 μm), column temperature = 120°C, injector temperature = 220°C, detector temperature = 250°C, and each injection volume was 1 μL.

The standard substances of SCFAs are as follows: acetic acid (A116165, Shanghai Aladdin Bio-Chem Technology Co., LTD, Shanghai, China), propionic acid (P110443, Aladdin), isobutyric acid (I103521, Aladdin), butyric acid (B110439, Aladdin), isovaleric acid (I108280, Aladdin), and valeric acid (V108269, Aladdin).

#### Fecal Untargeted Metabolomic Analysis

Aliquots of 100 mg of fecal samples were homogenized with 200 μL of ultra-pure water and mixed with 800 L methanol/acetonitrile (1:1, v/v) to remove the protein. After centrifugation (14,000 g, 20 min, 4°C), the supernatant was collected for analysis.

#### LC-MS Analysis and Data Processing

Analyses were performed using liquid chromatography (1,290 infinity liquid, Agilent) coupled with quadrupole time-of-flight (Triple TOF 6600, AB Sciex). For hydrophilic interaction liquid chromatography (HILIC) separation, samples were analyzed using a 2.1 × 100 mm ACQUIY UPLC BEH Amide 1.7 μm column (Waters, Ireland). Chromatographic conditions and Q-TOF mass spectrometry conditions were as outlined by Liu et al. (19), electrospray ionization (ESI) positive mode was used for detection.

The raw MS data were converted to MzXML format using ProteoWizard MSConvert and processed using the XCMS online package (version 1.20.1) for further processing. Metabolites were identified by accuracy mass (<25 ppm) and secondary spectral matching to retrieve a self-built database from the laboratory (Shanghai Applied Protein Technology). After data preprocessing by Pareto-scaling, multidimensional statistical analysis [orthogonal partial least squares discriminant analysis (OPLS-DA)] and Student's *t*-test analysis were performed. Significantly different metabolites were identified based on the combination of a statistically significant threshold of variable influence on projection (VIP) values obtained from the OPLS-DA model and two-tailed Student's *t*-test (*p*-value) from the raw data. VIP > 1.00 and *P* < 0.05 were considered as significantly different metabolites. VIP > 1.00 and 0.05 < *P* < 0.10 were regarded as differential metabolites.

### Statistical Analysis

Data were tested for normality using the Shapiro–Wilk test before statistical analysis. The growth performance, SCFA, and serum methyl metabolite profile between CON and MET piglets was assessed by independent-samples *t*-test. The individual sow was considered as the experimental unit for growth performance, one piglet per pen was used as the experimental unit for SCFA and methyl metabolite profile and microbial and metabolite analysis. Statistical analysis was performed on SPSS 24.0 software (SPSS Inc.). Correlations between altered metabolites screened from fecal metabonomic and perturbed gut microbe genera screened from 16S rDNA gene sequencing analysis were assessed by Spearman's correlation analysis. GraphPad Prism 8.0 (San Diego, CA, USA) was used to plot the images. Data are shown as means ± SEM. A value of *P* < 0.05 was considered statistical significance.

## Results

### Growth Performance and Serum SAM and Hcy Concentrations of Offspring Piglets

**Figure 1:**
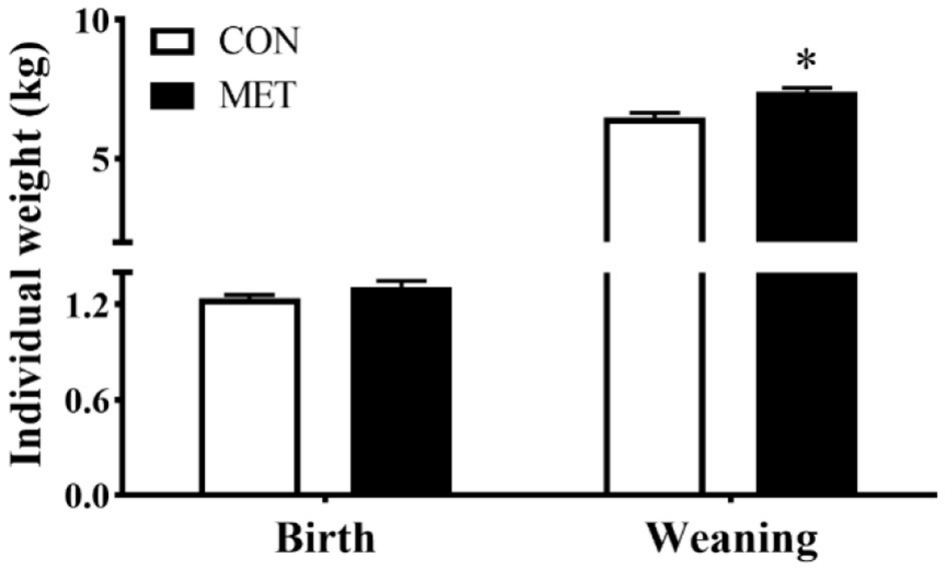
Effect of maternal methyl-donor micronutrient supplementation during gestation on birth weight and weaning weight of offspring piglets (*n* = 21). No difference was observed for litter size of lactating sows between treatments [data not shown, see reference (14)]. The litter of each sow was considered as the experimental unit to calculate the individual weight of piglets at birth and weanling. CON, the control group; MET, the methyl-donor micronutrients group. ^*^P < 0.05. Values are expressed as means ± standard error of mean (SEM).

There was no statistical difference in offspring birth weight between dietary treatments (*P* > 0.05). However, the body weight at weaning was greater for piglets from sows fed the MET vs. CON diet (*P* < 0.05) (Figure 1).

Maternal MET supplementation during gestation decreased serum Hcy concentration in the offspring at birth (*P* = 0.089, Table 1). Likewise, there was a significantly lower concentration of serum Hcy in the weaning offspring of the MET group compared with the CON group (*P* < 0.05). Moreover, the serum SAM concentration in piglets at birth and weaning was increased in the MET group (*P* = 0.061, *P* < 0.05, respectively).

**Table 1:** Effect of maternal MET supplementation during gestation on the concentration of S-adenosylmethionine (SAM) and homocysteine (Hcy) in the serum of newborn and weaning piglets (*n* = 6).

**Items**	**CON**	**MET**	***P*-value**
Birth			
SAM, μmol/mL	22.31 ± 0.86	24.55 ± 0.57	0.061
Hcy, nmol/mL	13.21 ± 0.24	11.90 ± 0.65	0.089
Weaning			
SAM, μmol/mL	19.40 ± 1.25	30.81 ± 2.81	0.002
Hcy, nmol/mL	13.15 ± 0.68	10.44 ± 0.98	0.037

*CON, the control group; MET, the methyl-donor micronutrients group. Data are reported as means ± standard error of mean (SEM)*.

### Fecal Microbiota Diversity

The alpha diversity indices of fecal microbiota in suckling piglets are shown in Table 2. The Shannon and Simpson indices of the fecal bacterial community were higher in 7-day piglets from sows fed the MET vs. CON diet (*P* < 0.05). The ACE, Chao1, observed species, and Simpson indices were higher in 21-day piglets from sows fed the MET vs. CON diet (*P* < 0.05).

**Table 2:** Effect of maternal methyl-donor micronutrient supplementation during gestation on the alpha diversity indices of fecal microbiota in suckling piglets (*n* = 6)^1^.

**Items**	**7 days old**		**14 days old**		**21 days old**	
	**CON**	**MET**	**CON**	**MET**	**CON**	**MET**
Shannon	5.23 ± 0.12^b^	5.69 ± 0.14^a^	5.64 ± 0.23	6.02 ± 0.21	5.71 ± 0.21	6.04 ± 0.21
Simpson	0.91 ± 0.01^b^	0.94 ± 0.01^a^	0.93 ± 0.02	0.95 ± 0.01	0.94 ± 0.01^b^	0.96 ± 0.01^a^
ACE	800.36 ± 21.96	879.75 ± 34.39	940.12 ± 35.24	913.13 ± 65.47	815.08 ± 23.23^b^	889.63 ± 18.09^a^
Chao1	797.97 ± 25.79	886.62 ± 40.21	936.55 ± 25.46	904.09 ± 65.59	806.62 ± 24.45^b^	882.62 ± 15.08^a^
Observed species	618.50 ± 12.41	679.17 ± 25.41	718.17 ± 22.17	733.00 ± 50.68	651.83 ± 11.12^b^	705.00 ± 12.88^a^

*CON, the control group; MET, the methyl-donor micronutrients group. At each time point, different letters indicate statistical difference in the same row (P <0.05). Values are expressed as means ± standard error of mean (SEM)*.

1 *n = 6: Six litters per group were selected, and one median-birth-weight piglet from each litter was sampled for feces collection at 7, 14, and 21 days*.

**Figure 2:**
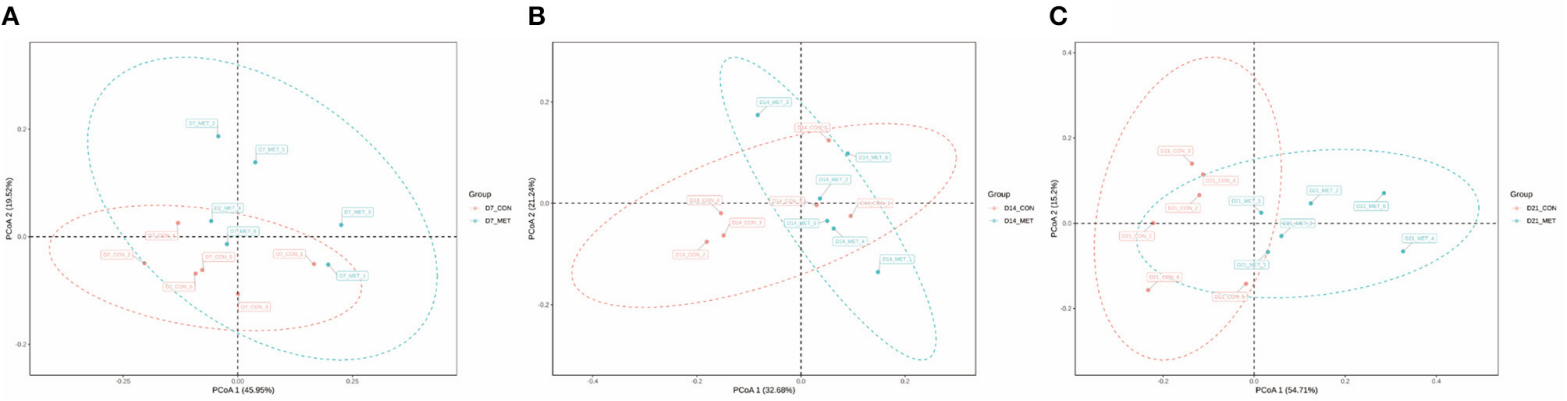
The PCoA analysis of the fecal microbial community in suckling piglets from sows fed the MET vs. CON diet. The PCoA plot was generated by the weighted UniFrac distances. Each dot represents an individual sample of piglet (*n* = 6). *n* = 6: Six litters per group were selected, and one median-birth-weight piglet from each litter was sampled for feces collection at 7 **(A)**, 14 **(B)**, and 21 days **(C)**. CON, the control group; MET, the methyl-donor micronutrients group.

Figure 2 presents the PCoA analysis of the fecal microbial community in suckling piglets from sows fed the MET vs. CON diet. PERMANOVA (Adonis procedure with 999 permutations) revealed distinct clustering patterns of feces microbiota in offspring piglets between two treatments at 7 days (*R*^2^ = 0.17, *P* < 0.05) and 21 days (*R*^2^ = 0.14, *P* < 0.05).

### Fecal Microbial Composition at Phylum and Genus Level

**Figure 3:**
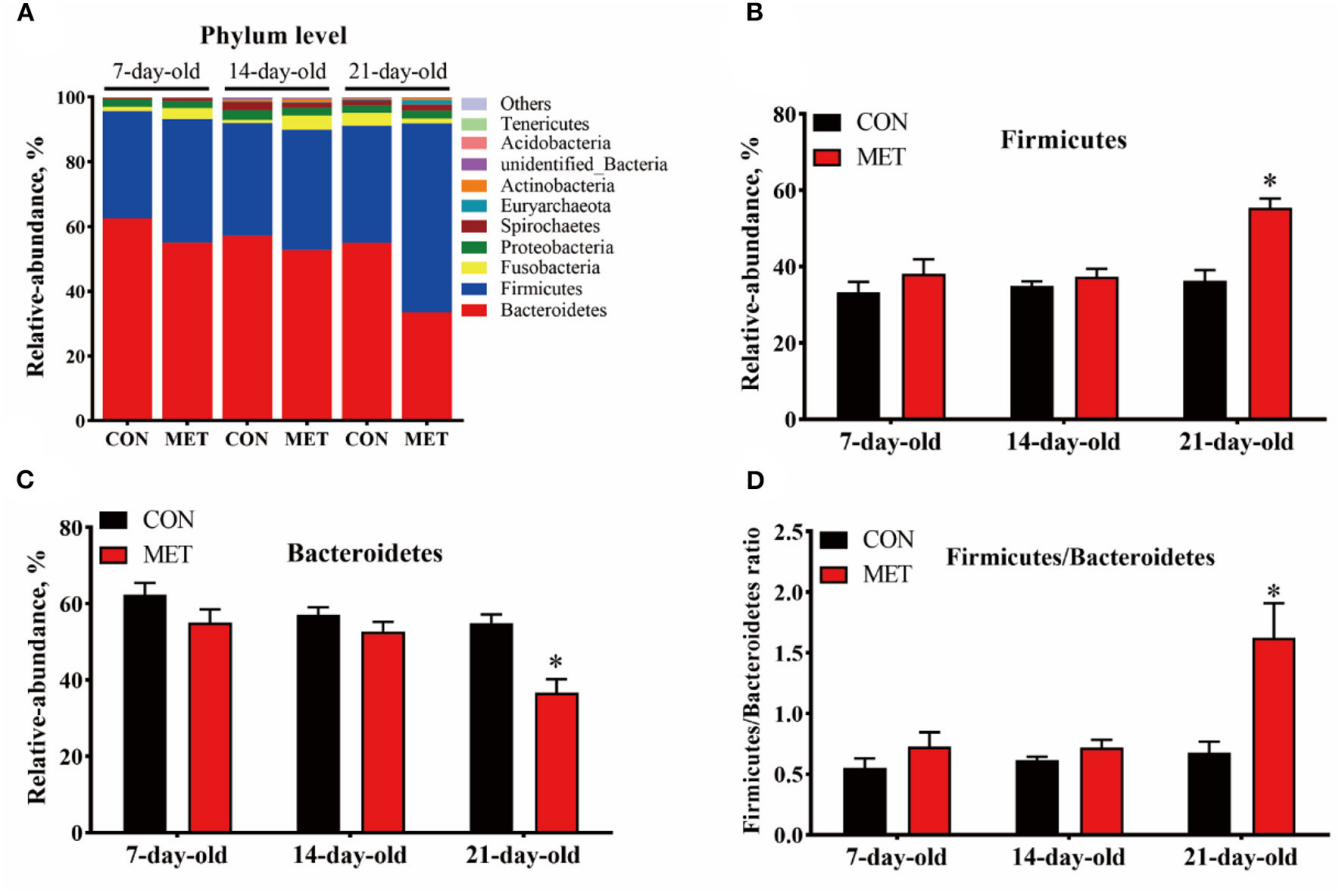
The composition of fecal microbiota in suckling piglets from sows fed the MET vs. CON diet at the phylum level. The relative abundance of the top 10 phyla **(A)** and the bacterial phyla differed **(B–D)** between suckling piglets from CON- and MET-fed sow. Each dot represents an individual piglet (*n* = 6), and ^*^ indicates *P* < 0.05. *n* = 6: Six litters per group were selected, and one median-birth-weight piglet from each litter was sampled for feces collection at 7, 14, and 21 days. CON, the control group; MET, the methyl-donor micronutrients group.

In both treatment groups, the Bacteroidetes, Firmicutes, Fusobacteria, and Proteobacteria were the dominant microbial phyla in the feces of offspring piglets, followed by Spirochaetes, Euryarchaeota, Actinobacteria, unidentified_Bacteria, Acidobacteria, and Tenericutes (Figure 3A). The relative abundance of Firmicutes and the Firmicutes/Bacteroidetes ratio were increased, and the relative abundance of Bacteroidetes were decreased in the feces of 21-day piglets from the MET group compared with the CON group (*P* < 0.05) (Figures 3B–D).

**Figure 4:**
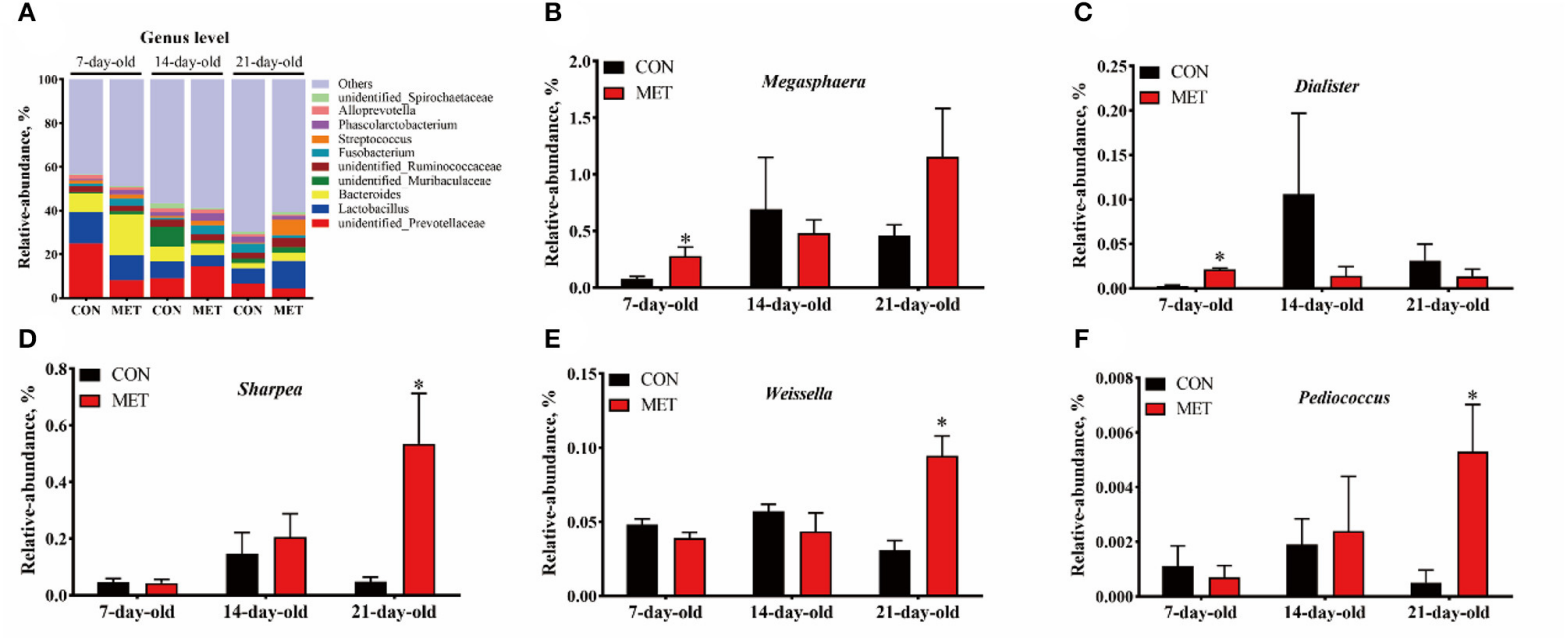
The composition of fecal microbiota in suckling piglets from sows fed the MET vs. CON diet at the genus level. The relative abundance of the top 10 genera **(A)** and the bacterial genera differed **(B–F)** between suckling piglets from CON- and MET-fed sow. Each dot represents an individual piglet (*n* = 6), and ^*^ indicates *P* < 0.05. *n* = 6: Six litters per group were selected, and one median-birth-weight piglet from each litter was sampled for feces collection at 7, 14, and 21 days. CON, the control group; MET, the methyl-donor micronutrients group.

The 10 most abundant genera in the feces of offspring piglets are *unidentified_Prevotellaceae, Lactobacillus, Bacteroides, unidentified_Muribaculaceae, unidentified_Ruminococcaceae, Fusobacterium, Streptococcus, Phascolarctobacterium, Alloprevotella, unidentified_Spirochaetaceae* (Figure 4A). The relative abundances of *Megasphaera* and *Dialister* were increased in the feces of 7-day piglets, and relative abundances of *Sharpea, Weissella*, and *Pediococcus* were increased in the feces of 21-day piglets when their mothers were fed the MET vs. CON diet (*P* < 0.05) (Figures 4B–F).

### Differential Fecal Microbial Communities

**Figure 5:**
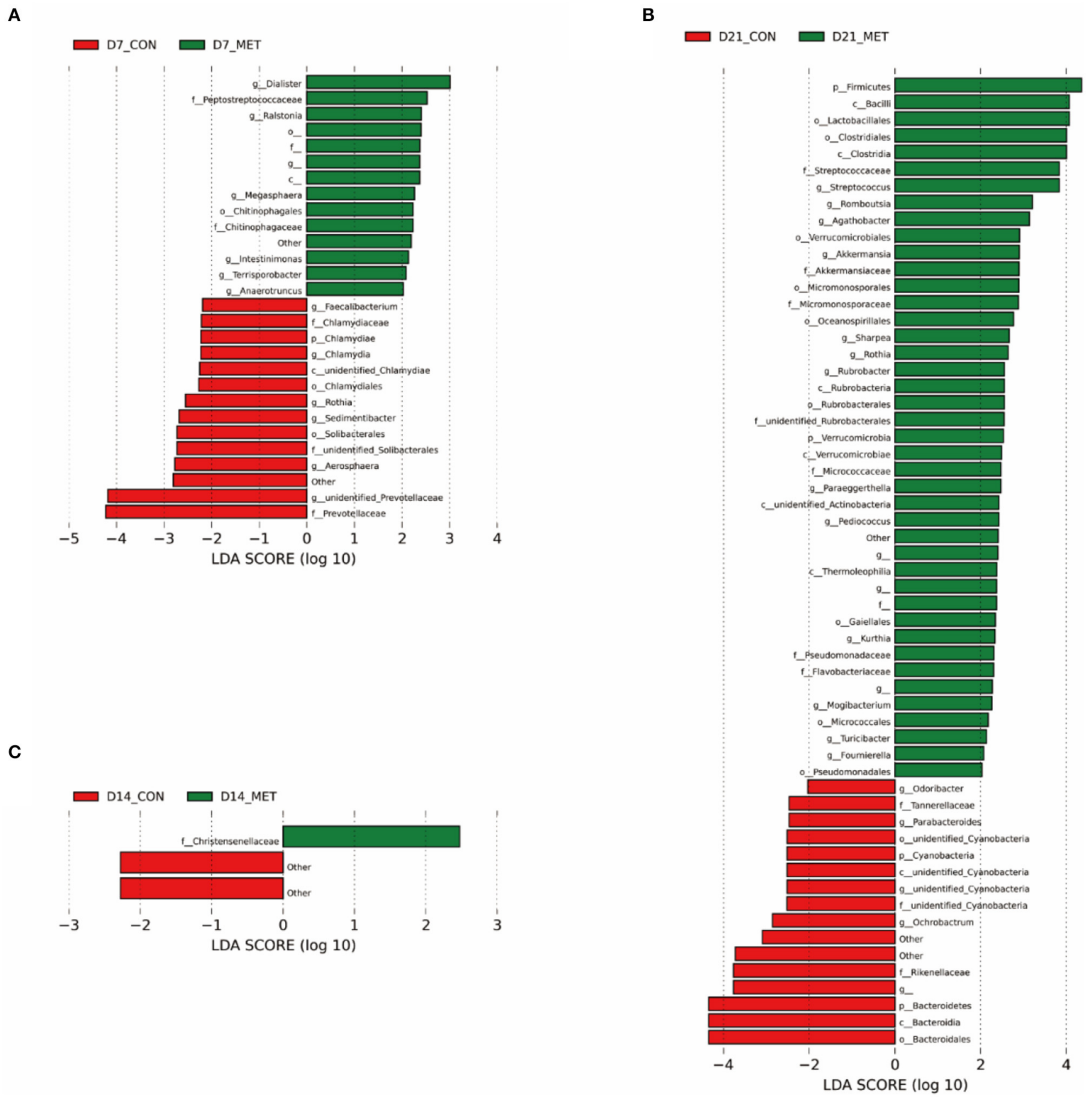
The LEfSe analysis of the fecal bacterial community in 7 **(A)**, 14 **(B)**, and 21- day **(C)** suckling piglets from sows fed the MET vs. CON diet (*n* = 6). The default parameter of the LDA score is 4. The bacteria that are not named in the figure are those that have not yet been named. *n* = 6: Six litters per group were selected, and one median-birth-weight piglet from each litter was sampled for feces collection at 7, 14, and 21 days. CON, the control group; MET, the methyl-donor micronutrients group; LDA, linear discriminant analysis; p_, phylum; c_, class; o_, order; f_, family; g_, genus.

Differences in the relative abundances of the microbial community components of CON and MET offspring were further analyzed by LEfSe. Compared with the CON group, including unknown bacteria, 14 microbial phlotypes were higher and 14 were lower in the 7-day suckling piglets from MET-fed sows (Figure 5A). At the age of 14 days, compared with the CON group, only one microbial phlotype was higher and two were lower in the MET offspring (Figure 5B). There were 42 higher and 16 lower microbial phlotypes in 21-day piglets from sows fed the MET vs. CON diet (Figure 5C).

Specific differentiated phylotypes were identified in 7-day suckling piglets from sows fed the MET vs. CON diet: increased genera *Dialister, Ralstonia, Megasphaera, Intestinimonas, Terrisporobacter, Anaerotrumcus* and families *Peptostretococcaceae, Chitinophagales* and order *Chitinophagaceae* (*P* < 0.05), decreased genera *Faecalibacterium, Rothia, Sedimentibacter, Aerosharea, unidentified-Prevotellaceae, Chlamydia* and families *Chlamydiaceae, Prevotellaceae, unidentified-Solibacterales* and order *Solibacterales, Chlamydiales* and class *unidentified-Chlamydiae*, phylum Chlamydiae (*P* < 0.05). At the age of 14 days, the suckling piglets from sows fed MET had significantly higher family *Christensenellaceae* (*P* < 0.05); at the age of 21 days, the suckling piglets from sows fed MET had increased genera *Streptococcus, Romboutsia, Agathobacter, Akkermansia, Sharpea, Rothia, Rubrobacter, Paraeggerthella, Pediococcus, Kurthia, Mogibacterium, Turicibacter, Foumirerlla* and families *Streptococcaceae, Akkermansiaceae, Micromonosporaceae, unidentified-Rubrobacterales, Micrococcaceae, Pseudomonadaceae, Flavobacteriaceae* and orders *Lactobacillales, Clostridiales, Verrucomicrobiales, Micromonosporaceae, Oceanospirillales, Rubrobacterales, Gaiellales, Micrococcales, Pseudomonadales* and classes *Bacilli, Clostridia, Rubrobacteria, Verrucomicrobia, unidentified-Actinobacteria, Thermoleophilia* and phyla Firmicutes, Verrucomicrobia (*P* < 0.05), decreased genera *Odoribater, Parabacteroides, unidentified-Cyanobacteria, Ochrobactrum* and families *Tanerellaceae, unidentified-Cyanobacteria, Rikenellaceae* and order *unidentified-Cyanobacteria, Bacteroidales* and classes *unidentified-Cyanobacteria, Bacteroidia* and phyla Cyanobacteria, Bacterioidetes (*P* < 0.05).

### Fecal SCFA Concentration

As displayed in Table 3, no statistical differences were observed in the fecal SCFA concentration of 7-day piglets between maternal dietary treatments (*P* > 0.05). However, the concentration of acetate, butyrate, and total SCFA were increased in the feces of 14-day piglets from sows fed the MET vs. CON diet (*P* < 0.05). Besides this, maternal MET supplementation increased the concentration of acetate, propionate, isobutyrate, butyrate, isovalerate, valerate, valerate, and total SCFA in the feces of 21-day piglets (*P* < 0.05).

**Table 3:** Effect of maternal methyl-donor micronutrient supplementation during gestation on the concentration of short-chain fatty acids (SCFA) in feces of suckling piglets (mg/g) (*n* = 6)^1^.

**Items**	**7 days old**		**14 days old**		**21 days old**	
	**CON**	**MET**	**CON**	**MET**	**CON**	**MET**
Acetate	1.74 ± 0.14	2.19 ± 0.19	1.95 ± 0.14^b^	2.67 ± 0.12^a^	2.17 ± 0.14^b^	2.9 ± 0.28^a^
Propionate	0.83 ± 0.11	1.07 ± 0.10	0.99 ± 0.08	1.24 ± 0.13	1.15 ± 0.12^b^	1.56 ± 0.10^a^
Isobutyrate	0.16 ± 0.03	0.18 ± 0.03	0.17 ± 0.02	0.24 ± 0.03	0.16 ± 0.02^b^	0.40 ± 0.07^a^
Butyrate	0.52 ± 0.03	0.55 ± 0.06	0.59 ± 0.08^b^	0.96 ± 0.11^a^	0.60 ± 0.05^b^	1.86 ± 0.28^a^
Isovalerate	0.39 ± 0.07	0.40 ± 0.07	0.38 ± 0.05	0.50 ± 0.06	0.41 ± 0.04^b^	1.11 ± 0.16^a^
Valerate	0.24 ± 0.03	0.36 ± 0.08	0.27 ± 0.05	0.43 ± 0.09	0.25 ± 0.03^b^	0.51 ± 0.05^a^
The ratio of acetate to propionate	2.29 ± 0.31	2.11 ± 0.09	2.01 ± 0.11	2.33 ± 0.34	1.94 ± 0.13	1.88 ± 0.19
Total SCFA	3.87 ± 0.36	4.79 ± 0.47	4.34 ± 0.40^b^	6.03 ± 0.24^a^	4.75 ± 0.37^b^	8.33 ± 0.86^a^

*SCFA, short-chain fatty acid; CON, the control group; MET, the methyl-donor micronutrients group. At each time point, different letters indicate statistical difference in the same row (P <0.05). Values are expressed as means ± standard error of mean (SEM)*.

1 *n = 6: Six litters per group were selected, and one median-birth-weight piglet from each litter was sampled for feces collection at 7, 14, and 21 days*.

### Fecal Metabolic Profiling

**Figure 6:**
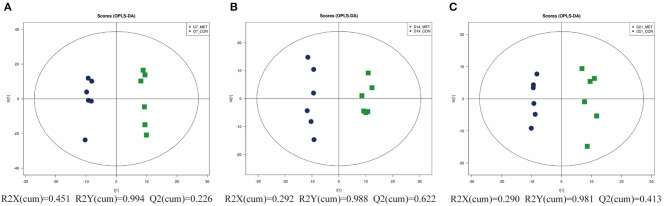
The OPLS-DA analysis (orthogonal partial least squares discriminant analysis) of fecal microbial metabolites in 7 **(A)**, 14 **(B)**, and 21- day **(C)** suckling piglets from sows fed the MET vs. CON diet (*n* = 6). *n* = 6: Six litters per group were selected, and one median-birth-weight piglet from each litter was sampled for feces collection at 7, 14, and 21 days. CON, the control group; MET, the methyl-donor micronutrients group.

As graphed in Figure 6, the separation in positive ionization mode between the two groups by the OPLS-DA method was evaluated. OPLS-DA plots of the fecal metabolomics data show a clear separation with no overlap for each group, the parameters for the explanatory and predictive values of the intercepts (R2, Q2), which were stable and good to fitness and prediction (Supplementary Figure 1).

### Differential Metabolites and Bioinformatics Analysis

**Figure 7:**
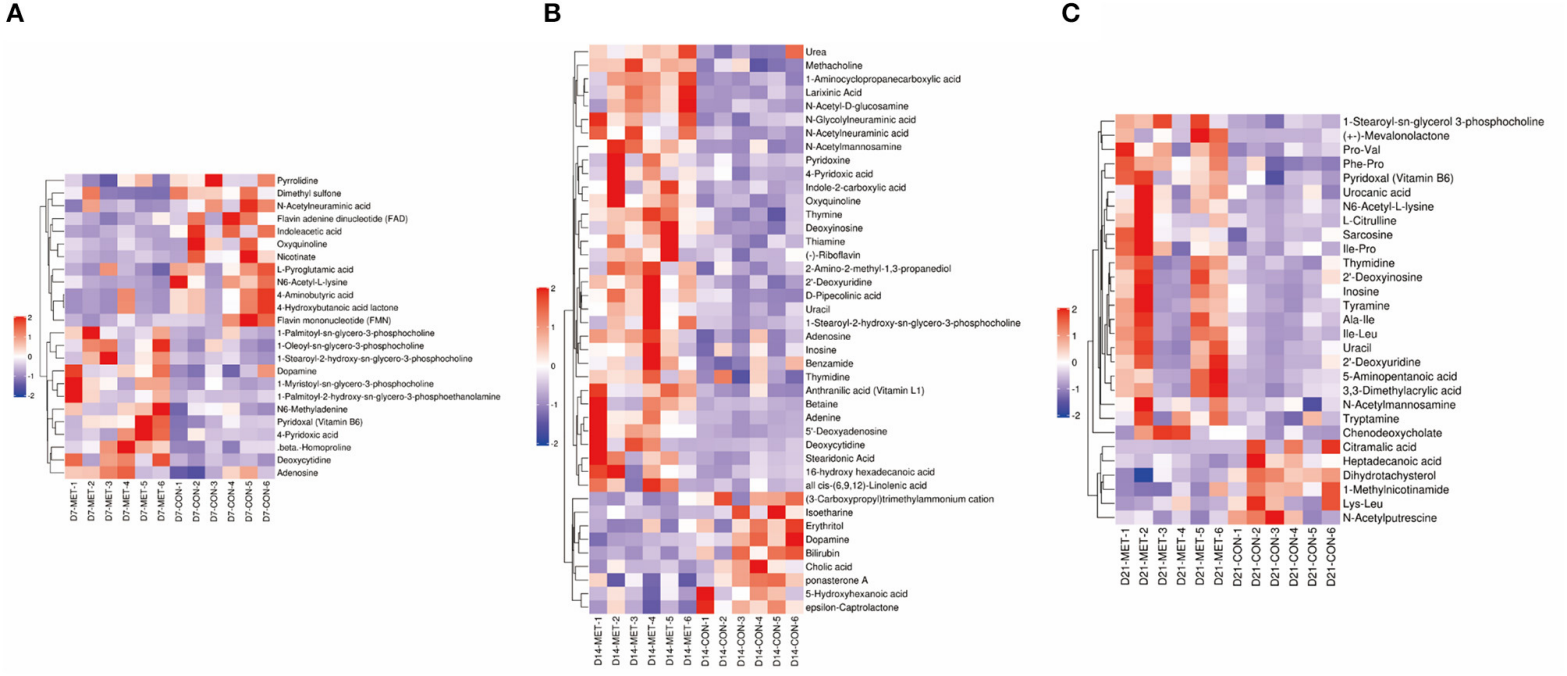
The hierarchical clustering of fecal metabolic profile in 7 **(A)**, 14 **(B)**, and 21- day **(C)** suckling piglets from sows fed the MET vs. CON diet (*n* = 6). The color scale ranges from saturated purple (−2) to blank (0) to saturated red (2). Red and purple color represent increased and decreased metabolites, respectively. X axis: sample code, Y axis: metabolite name. *n* = 6: Six litters per group were selected, and one median-birth-weight piglet from each litter was sampled for feces collection at 7, 14, and 21 days. CON, the control group; MET, the methyl-donor micronutrients group.

Based on the VIP of the OPLS-DA model and the *p*-value of the Student's *t*-test, the biologically important difference metabolites were mined. Including significantly differential and differential metabolites, a total of 24 differentiated metabolites were identified from two data sets in 7-day suckling piglets (Figure 7A), 42 differentiated metabolites were identified from two data sets in 14-day suckling piglets (Figure 7B), and 29 differentiated metabolites were identified from two data sets in 21-day suckling piglets (Figure 7C). We subsequently analyzed the significantly metabolite differences in the Kyoto Encyclopedia of Genes and Genomes (KEGG) pathway database (http://www.genome.jp/kegg/) to pathways in offspring that may have been influenced by maternal MET during gestation. These findings revealed that the potential biomarkers contribute to various processes, including biosynthesis of amino acids, amino acid metabolism, pyrimidine metabolism, purine metabolism, amino sugar and nucleotide sugar metabolism, bile secretion, thiamine metabolism, vitamin B6 metabolism, riboflavin metabolism, nicotinate and nicotinamide metabolism, sulfur metabolism, and glutathione metabolism (Tables 4–6).

**Table 4:** The significantly changed fecal metabolites in 7-day suckling piglets from sows fed the MET vs. CON diet (*n* = 6)^1^.

**Metabolites**	**Fold change (MET vs. CON)**	***P*-value**	**VIP**	***m/z***	**Pathway**
Deoxycytidine	5.187	0.007	1.397	455.188	Pyrimidine metabolism
Indoleacetic acid	0.191	0.008	2.425	176.069	Tryptophan metabolism
Pyridoxal (Vitamin B6)	1.770	0.022	1.053	168.064	Vitamin B6 metabolism
Flavin adenine dinucleotide (FAD)	0.589	0.040	1.247	786.165	Riboflavin metabolism
Flavin mononucleotide (FMN)	0.418	0.049	1.125	457.110	Riboflavin metabolism
Nicotinate	0.191	0.038	1.364	124.041	Nicotinate and nicotinamide metabolism
N6-acetyl-l-lysine	0.403	0.009	2.201	189.122	Lysine degradation
Dimethyl sulfone	0.433	0.035	1.197	226.986	Sulfur metabolism
L-pyroglutamic acid	0.619	0.045	1.593	190.070	Glutathione metabolism
Beta-homoproline	3.459	0.008	1.007	130.085	Other
1-Myristoyl-sn-glycero-3-phosphocholine	3.068	0.017	1.150	468.307	Other
1-Oleoyl-sn-glycero-3-phosphocholine	1.912	0.028	2.715	522.355	Other
1-Stearoyl-2-hydroxy-sn-glycero-3-phosphocholine	4.347	0.036	1.757	506.358	Other

*CON, the control group; MET, the methyl-donor micronutrients group. VIP variable importance for the projection, m/z mass to charge ratio*.

1 *n = 6: Six litters per group were selected, and one median-birth-weight piglet from each litter was sampled for feces collection at 7 days*.

**Table 5:** The significantly changed fecal metabolites in 14-day suckling piglets from sows fed the MET vs. CON diet (*n* = 6)^1^.

**Metabolites**	**Fold change (MET vs. CON)**	***P*-value**	**VIP**	***m/z***	**Pathway**
1-Aminocyclopropanecarboxylic acid	4.947	0.000	1.449	84.043	Biosynthesis of amino acids
Anthranilic acid (Vitamin L1)	2.160	0.005	2.151	138.054	Biosynthesis of amino acids
Thymine	2.357	0.001	4.485	127.049	Pyrimidine metabolism
2'-Deoxyuridine	3.223	0.002	1.044	229.08	Pyrimidine metabolism
Uracil	2.675	0.024	1.155	113.033	Pyrimidine metabolism
Urea	1.450	0.043	1.137	61.039	Pyrimidine metabolism
Deoxycytidine	7.633	0.048	1.482	455.188	Pyrimidine metabolism
Adenosine	2.25	0.007	1.385	268.103	Purine metabolism
Adenine	3.111	0.011	3.814	136.061	Purine metabolism
Deoxyinosine	2.731	0.016	1.182	253.092	Purine metabolism
N-Acetylmannosamine	3.443	0.002	1.750	204.086	Amino sugar and nucleotide sugar metabolism
N-Acetylneuraminic acid	5.873	0.005	2.319	310.112	Amino sugar and nucleotide sugar metabolism
N-Acetyl-D-glucosamine	4.727	0.006	1.521	443.186	Amino sugar and nucleotide sugar metabolism
N-Glycolylneuraminic acid	2.558	0.007	1.172	326.107	Amino sugar and nucleotide sugar metabolism
Dopamine	0.508	0.005	1.937	136.074	Bile secretion
Bilirubin	0.228	0.007	5.162	585.269	Bile secretion
Cholic acid	0.310	0.047	1.652	426.319	Bile secretion
Thiamine	2.299	0.014	4.665	265.112	Thiamine metabolism
4-Pyridoxic acid	2.672	0.043	2.394	184.059	Vitamin B6 metabolism
Betaine	6.706	0.028	7.651	118.086	Glycine, serine and threonine metabolism
Erythritol	0.367	0.044	2.214	164.090	ABC transporters
Methacholine	2.472	0.001	1.141	160.132	Other
Larixinic acid	4.992	0.002	3.898	144.064	Other
Oxyquinoline	3.131	0.002	1.555	146.059	Other
D-Pipecolinic acid	5.572	0.003	7.555	130.086	Other
Epsilon-captrolactone	0.496	0.004	2.425	156.101	Other
5'-Deoxyadenosine	4.760	0.004	7.327	252.109	Other
(3-Carboxypropyl) Trimethylammonium cation	0.285	0.013	3.409	146.116	Other
2-Amino-2-methyl-1,3-propanediol	2.512	0.014	1.886	70.064	Other
Ponasterone A	0.439	0.015	1.376	482.345	Other
Indole-2-carboxylic acid	5.840	0.021	3.140	162.054	Other
Stearidonic acid	2.479	0.024	2.942	277.215	Other
1-Stearoyl-2-hydroxy-sn-glycero-3-phosphocholine	3.972	0.028	1.829	506.356	Other

*CON, the control group; MET, the methyl-donor micronutrients group. VIP variable importance for the projection, m/z mass to charge ratio*.

1 *n=6: Six litters per group were selected, and one median-birth-weight piglet from each litter was sampled for feces collection at 14 days*.

**Table 6:** The significantly changed fecal metabolites in 21-day suckling piglets from sows fed the MET vs. CON diet (*n* = 6)^1^.

**Metabolites**	**Fold change (MET vs. CON)**	***P*-value**	**VIP**	***m/z***	**Pathway**
Sarcosine	2.169	0.035	1.415	150.075	Biosynthesis of amino acids
1-Methylnicotinamide	0.457	0.017	2.128	137.070	Bile secretion
Pyridoxal (Vitamin B6)	2.056	0.012	1.844	168.064	Vitamin B6 metabolism
N6-Acetyl-L-lysine	3.092	0.048	1.020	189.122	Lysine degradation
Dihydrotachysterol	0.578	0.000	1.346	465.320	Other
Phe-Pro	2.199	0.001	2.128	263.138	Other
1-Stearoyl-sn-glycerol 3-phosphocholine	3.303	0.006	2.147	568.339	Other
Pro-Val	2.994	0.020	2.184	215.138	Other
Ile-Leu	2.964	0.033	1.547	245.186	Other
3,3-Dimethylacrylic acid	2.520	0.034	2.222	101.059	Other
Ala-Ile	3.760	0.044	2.163	203.138	Other
Ile-Pro	2.184	0.045	3.157	229.154	Other
(+-)-Mevalonolactone	3.800	0.045	1.183	148.095	Other

*CON, the control group; MET, the methyl-donor micronutrients group. VIP, variable importance for the projection, m/z mass to charge ratio*.

1 *n = 6: Six litters per group were selected, and one median-birth-weight piglet from each litter was sampled for feces collection at 21 days*.

Specific differentiated metabolites were identified in 7-day suckling piglets of CON-fed and MET-fed sows: Deoxycytidine closely related to pyrimidine metabolism was increased 5.187-fold in MET piglets compared with CON piglets (*P* < 0.05). Pyridoxal closely related to vitamin B6 metabolism was increased 1.770- fold in MET compared with the CON group (*P* < 0.05). Indoleacetic acid closely related to tryptophan metabolism was decreased 0.191-fold in MET piglets compared with CON piglets (*P* < 0.05). Riboflavin metabolism (flavin adenine dinucleotide and flavin mononucleotide) were decreased 0.59- and 0.42-fold in MET piglets compared with CON piglets (*P* < 0.05). In the 14-day suckling piglets, critical metabolites (1-aminocyclopropanecarboxylic acid, anthranilic acid) closely related to biosynthesis of amino acids were increased by 4.95- and 2.16-fold in the MET group compared with the CON group, respectively (*P* < 0.05). Thymine, 2'-deoxyuridine, uracil, urea, and deoxycytidine, which are involved in pyrimidine metabolism, were increased in the MET compared with the CON group by 2.36-, 3.22-, 2.68-, 1.45-, and 7.63-fold, respectively (*P* < 0.05), and concentrations of adenosine, adenine, and deoxyinosine, which are involved in purine metabolism, were increased in the MET compared with the CON group by 1.39-, 3.81-, and 1.18-fold, respectively (*P* < 0.05). Critical metabolites (dopamine, bilirubin, and cholic acid) closely related to bile secretion were decreased in the MET group. In addition, critical metabolites closely related to amino acid and vitamin B6 metabolism were increased in the MET group compared with the CON group (*P* < 0.05). In the 21-day suckling piglets,: critical metabolites related to biosynthesis of amino acids, amino acid metabolism, and vitamin B6 metabolism, were increased in the MET group compared with CON group although bile secretion was decreased in MET piglets (*P* < 0.05).

### Correlation Analysis for Differential Metabolites and Microbes

**Figure 8:**
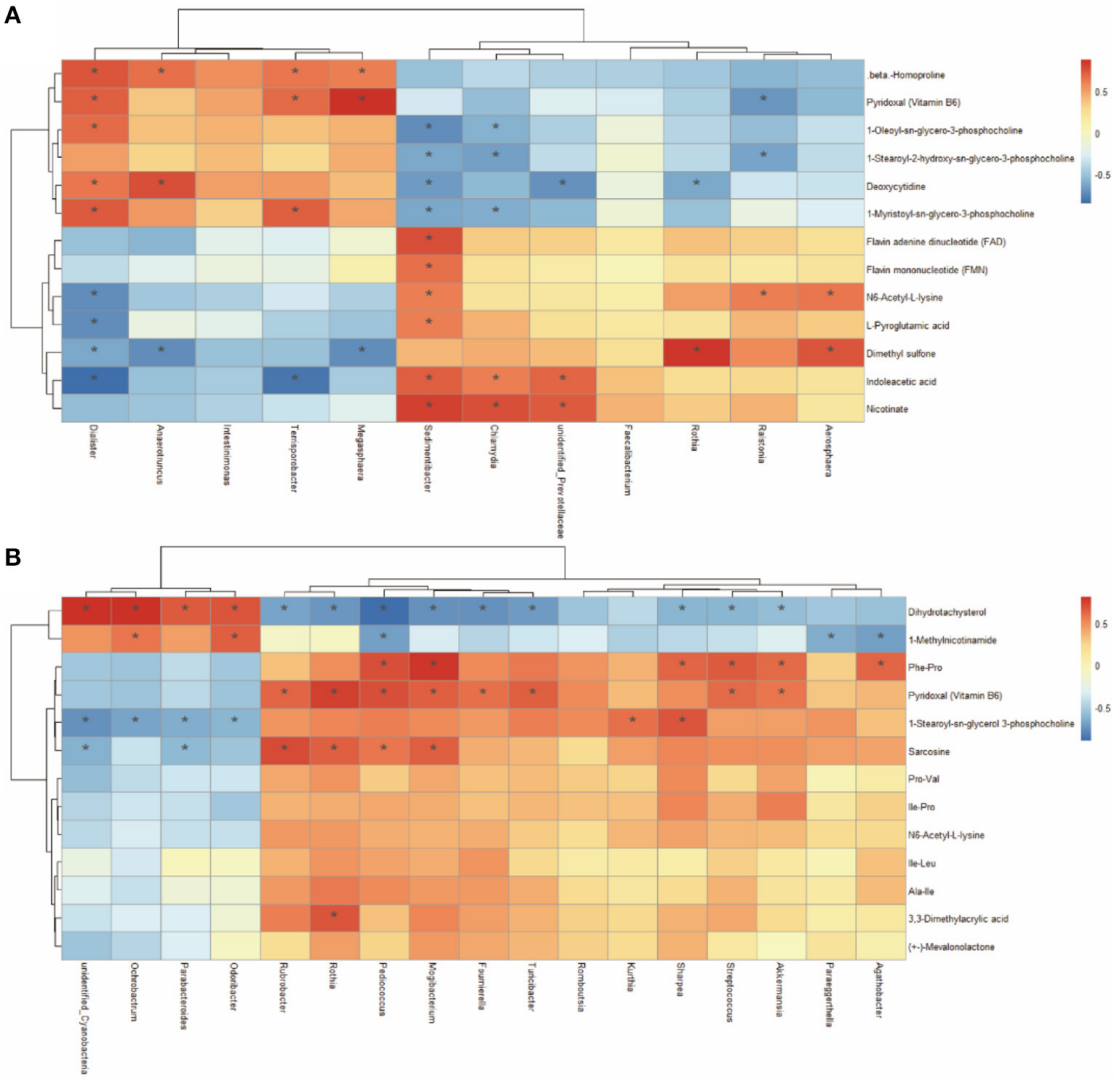
Spearman correlation analysis between genera and metabolite concentrations in 7 **(A)** and 21- day **(B)** suckling piglets from sows fed the MET vs. CON diet (*n* = 6). ^*^Asterisks indicate significant correlations between genera and metabolite concentrations. Cells are colored based on the Spearman correlation coefficient between the significantly altered genera and metabolites; red represents a significantly positive correlation, blue represents a significantly negative correlation (*P* < 0.05), and white represents no significant correlation (*P* > 0.05). *n* = 6: Six litters per group were selected, and one median-birth-weight piglet from each litter was sampled for feces collection at 7, 14, and 21 days.

A Spearman's correlation matrix was generated to explore the correlation between the bacterial genera and candidate compounds that were affected by maternal nutrition. As shown in Figure 8, significant associations could be identified between the gut microbial and the altered metabolite profiles from 7- and 21-day suckling piglets. In the 21-day suckling piglets (Figure 8A), the correlation analysis revealed that deoxycytidine was positively correlated with the genera *Dialister* and *Anaerotrumcus* and negatively associated with the genera *Rothia, Sedimentibacter, and unidentified-Prevotellaceae* (*P* < 0.05). Indoleacetic acid was negatively associated with the genera *Dialister* and *Terrisporobacter* and positively correlated with the genera *Sedimentibacter, unidentified-Prevotellaceae*, and *Chlamydia* (*P* < 0.05). The genera *Dialister, Megasphaera*, and *Terrisporobacter* were positively correlated with vitamin B6 (*P* < 0.05). In addition, the genus *Sedimentibacter* was positively correlated with flavin adenine dinucleotide and flavin mononucleotide (*P* < 0.05). In the 21-day suckling piglets (Figure 8B), sarcosine was negatively associated with the genera *Parabacteroides* and *unidentified-Cyanobacterir* and positively correlated with the genera *Rothia, Rubrobacter, Pediococcus*, and *Mogibacterium* (*P* < 0.05). 1-Methylnicotinamide was negatively associated with the genera *Agathobacter, Paraeggerthella*, and *Pediococcus* and positively correlated with the genera *Odoribater* and *Ochrobactrum* (*P* < 0.05). Nine genera, including *Streptococcus, Akkermansia, Rothia, Rubrobacter, Pediococcus, Mogibacterium, Turicibacter*, and *Foumirerlla* were positively correlated with vitamin B6 (*P* < 0.05).

## Discussion

In a previous study, we found no differences in reproductive performance of sows between CON and MET; however, we observed that the offspring of sows fed with the MET diet grew faster than control offspring, and maternal MET exposure can promote skeletal muscle differentiation and maturity and improve the skeletal muscle mass of weaning piglets (14). Previous research reports the intestinal tract of the monogastric animal plays a crucial role in nutrient digestion and absorption and maintains the barrier function against malignant pathogens and antigens (20). In addition, gut microbiota contributes to muscle mass and muscle fiber types through the gut–muscle axis (21). Thus, in the present study, we collected fresh fecal samples from piglets at 7, 14, and 21 days and further analyzed the effects of MET on intestine microbiota colonization and metabolism in offspring. Our findings show that the establishment of the suckling piglets' gut microbiota was strongly affected by maternal MET, and the MET offspring have a higher growth performance, which is concomitant with alterations in the intestinal microbiota alpha diversity, microbiota composition, SCFA, and the biosynthesis of amino acids. Furthermore, we found associations between the differentially abundant genera and metabolites in the CON and MET groups.

### Establishment of the Gut Microbial Community in Suckling Piglets

The gut microbiota diversity is highly related to host health and metabolic capacity (22). The low diversity of intestinal microbiota is considered a mark of intestinal dysbiosis, which may lead to autoimmune diseases, inflammatory bowel diseases, diarrhea, and metabolic diseases (23, 24). Increased bacterial richness and diversity is a marker of the establishment of intestinal microbiota (10). Our findings show that bacterial richness and diversity were increased in 7-day piglets from the MET sows compared with those from the CON sows. In addition, bacterial richness and uniformity were significantly higher in MET offspring at 21 days of age, suggesting that MET offspring had a more diversified intestinal microbiota. However, there was no difference in the microbiota diversity of 14-day piglets, this is probably because the gut microbial community is unstable and in a stage of rapid change. The bacterial communities were dominated by Firmicutes and Bacteroidetes in agreement with a previous study (25, 26). The increase in Firmicutes phylum is considered a marker of the establishment of intestinal microbiota (1, 27, 28). Previous studies found that the F/B is proportional to body weight because the increase in Firmicutes is closely related to energy intake (29, 30), and the relative abundance of Bacteroidetes is associated with degradation of proteins and carbohydrates (31). In our study, the microbiota of MET piglets showed an increase in F/B over time, characterized by increases in Firmicutes abundance. This suggesting that the intestinal microbiota of MET offspring may be more efficient than CON offspring at extracting energy from their diet, and consequently, they gain more weight.

### Differences in the Gut Microbiota Between CON and MET Offspring During Suckling Period

As is previously reported, an increase in methionine intake from the diet would lead to an increased amount of methionine in the lumen and provide an excellent fuel source for the rapid proliferation of bacteria (32). However, a lack of methyl donors can affect the differentiation and barrier functions of the small intestine and increase the concentration of Hcy, which subsequently promotes oxidative stress and activates interrelated pro-inflammatory mechanisms, ultimately aggravating inflammatory bowel disease in rats (13, 33). MET supplementation through the diet limits pathobiont colonization of the gut via inducing a specific intestinal micro-environment (11). When the harmful bacteria decrease, the beneficial bacteria may increase relatively. In our study, maternal MET supplementation during gestation decreased serum Hcy concentration in the offspring. Moreover, we identified that MET offspring at 7 days had a significantly higher abundance of *Dialister* and *Megasphaera* in the feces than CON offspring, and MET offspring at 21 days had a significantly higher abundance of *Romboutsia, Akkermansia, Sharpea, Turicibacter weissella*, and *pediococcus* in the feces than CON offspring. *Dialister* populations utilize succinate and produce propionate, *Megasphaera* can convert lactic acid to propionate via the acrylate pathway (34), and the *Sharpea* is beneficial to lactate production (35). Previous studies indicate that *Akkermansia* is linked with improved metabolic parameters; hosts with low *Akkermansia* content are susceptible to obesity, inflammation, and type 2 diabetes (36). As putatively beneficial gut microbiota, *Akkermansia* reinforces the gut barrier function and eventually reduces plasma lipopolysaccharide (37, 38). Previous studies also show that high relative abundances of *Romboutsia* are associated with decreased risk for infection in kidney transplant recipients (39), the *Turicibacter* might play a role in inflammatory bowel diseases and was proportional in concentration to that of butyric acid (40, 41). *Weissella* and *pediococcus* are recognized probiotic genera, which play a role in improving the health of the host (42, 43). Our data show that the relative abundance of beneficial bacteria is increased in MET piglets, which could play an inhibitory role in the growth of intestinal pathogenic bacteria and relieve metabolic diseases and intestinal inflammation (44, 45).

### Changes in Feces SCFA Between CON and MET Offspring During Suckling Period

Our results indicate that maternal MET supplementation during gestation increased the concentrations of individual and total SCFAs of offspring at 21 days of age. As for the reason for the increased SCFAs in the feces of suckling piglets, a previous study reports that acetate, propionate, and butyrate were formed from cysteine, whereas the main products of methionine metabolism were propionate and butyrate (46). Supplementing DL-MHA (a methionine substitute) in the diet increased the concentration of acetic acid, valeric acid, and total SCFAs in the cecum of piglets (47). Due to the sparing effect between methionine and cysteine, the supplementation of MET may lead to quantities of residual Cys in the gut to produce the SCFAs. In addition, the relative abundance of SCFA-producing *Dialister, Megasphaera, Turicibacter*, and *Sharpea* were increased in the MET group, which may also explain the increased production of SCFAs in the intestine. As the main source of energy for epithelial cells, SCFA can increase the proliferation of intestinal tissues (48, 49), butyrate can decrease intestinal inflammation, and as it enhances the intestinal barrier function (50), propionate can be used by hepatocyte cells of the liver for gluconeogenesis (51). That beneficial bacteria were increased in the MET offspring may be associated with the increase of SCFAs, which destroy microbial pathogens (52). However, no alteration in the concentrations of individual or total SCFAs was observed when the offspring piglets were 7 days old. Although the butyric- and lactic-producing bacteria were increased, the total intestinal microbiota of 7-day piglets was relatively low, and the amount of SCFA produced was also low, so the difference is not significant.

### Changes in Feces Metabolism Between CON and MET Offspring During Suckling Period

Maternal nutrition is clearly an important determinant of offspring gut microbiota, which has in turn linked with host metabolism and health (53, 54). The increase in BW at weaning might result from the increased metabolism of biosynthesis of amino acids, pyrimidine, and purine, which are positively correlated with growth. Previously, it was reported that amino acids not only serve as substances for synthesis of tissue proteins, but also as substrates for the synthesis of many low-molecular-weight substances (55). In our study, we found that maternal exposure to MET increased the concentrations of amino acid metabolism (including anthranilic acid, 1-aminocyclopropanecarboxylic acid, and sarcosine) in the offspring at 14 or 21 days. Meanwhile, the concentrations of metabolites involved in purine and pyrimidine metabolism increased, which indicated an enhanced function in nucleic acid metabolism via one-carbon metabolism (56) mainly because folate metabolism provides building blocks (10-formyl-tetrahydrofolate, methylene-tetrahydrofolate, respectively) for purine and pyrimidine synthesis. Furthermore, the amino and nucleotide sugars were increased in 14-day offspring of MET-supplemented sows. Notably, bile acid metabolites (cholic acid, dopamine, bilirubin, and 1-methylnicotinamide) were increased in 14- or 21-day offspring of sows in the CON group. The increase in bile acid metabolites could be caused by reduced reabsorption in the intestine (57). Bile acids (BAs) play an important role in the intestines, facilitating fat digestion and the absorption of lipids and liposoluble vitamins. Approximately 90% of bile acids return from the intestinal cavity to the liver via the portal vein (58). High concentrations of bile acid are toxic to mammalian cells (59). Cholic acid, as one of the BAs produced by the liver, is involved in the primary BA biosynthesis pathway (60). The elevation of cholic acid is a potential biomarker of liver injury, and extra cholic acid may partly lead to an increased risk of inflammation (61, 62). Total bilirubin is another indirect marker of liver function. Therefore, the decreased levels of cholic acid and bilirubin suggest that maternal exposure to MET during pregnancy may improve liver function of offspring.

The maternal metabolic status during gestation exerts a significant influence on the infant microbiota at the beginning of life (63). Maternal MET exposure can alter serum one-carbon metabolism of sows (14) and offspring. One-carbon metabolism participates in the synthesis of nucleotides, proteins, and lipids by integrating glucose, amino acid status, and vitamins in suckling piglets (8). In addition, there is increasing evidence that microbiota-derived metabolites act as key factors regulating animal metabolism, growth, and development (10). In this study, Spearman's correlation revealed an association between the abundance of specific bacterial genera and metabolites that were significantly influenced by maternal exposure to MET. Particularly, the VB6 and sarcosine were positively correlated with the genera *Rothia, Rubrobacter, Pediococcus*, and *Mogibacterium*. Altogether, the establishment of a gut microbial community and metabolic homeostasis could be a major underlying factor that induces improved growth and development of MET suckling piglets.

## Conclusion

Collectively, maternal methyl-donor micronutrient addition altered gut microbiota and the fecal metabolic profile, resulting in an improved weaning weight of offspring piglets.

## Data Availability Statement

The raw sequences used in this study were stored on the Sequence Read Archive (SRA) of NCBI, and the SRA accession number is PRJNA694233.

## Ethics Statement

The animal study was reviewed and approved by the Animal Care and Use Committee of Jiangxi Agricultural University (Ethic Approval Code: JXAUA01).

## Author Contributions

JY and TZ designed the study. QH, LJ, and JH performed the animal feeding experiment and sample analysis. FX assisted with SCFAs analysis. QH collected the data and wrote the manuscript. ZW and JC finalized the manuscript. All authors agree to be accountable for the content of the work.

## Conflict of Interest

The authors declare that the research was conducted in the absence of any commercial or financial relationships that could be construed as a potential conflict of interest.
