# Supplementary material for: Methyl-Donor Micronutrient for Gestating Sows: Effects on Gut Microbiota and Metabolome in Offspring Piglets
Source: Front Nutr. 2021 Jun 7;8:675640. doi: 10.3389/fnut.2021.675640 (PMC8215270; doi:10.3389/fnut.2021.675640)
Supplement: Supplementary file 1 [file Data_Sheet_1.docx]

**Table S1.** Ingredients and nutrient composition of diets for the pregnant sows

| Items | CON | MET |
| --- | --- | --- |
| Ingredients, % |  |  |
| Corn | 55.00 | 55.00 |
| Soybean hull | 10.00 | 10.00 |
| Rice bran | 10.00 | 10.00 |
| Expanded soybean | 8.00 | 8.00 |
| Soybean meal | 13.00 | 13.00 |
| Dicalcium phosphate | 1.70 | 1.70 |
| Limestone | 0.20 | 0.20 |
| NaCl | 0.30 | 0.30 |
| Lysine monohydrochloride, 98.5% | 0.05 | 0.05 |
| Choline chloride, 60% | 0.17 | 0.17 |
| Minerals^a^ | 0.40 | 0.40 |
| Vitamins^a^ | 0.05 | 0.05 |
| Methyl donnor^b^ | 0 | 1.11 |
| Filler^c^ | 1.13 | 0.02 |
| Total | 100.00 | 100.00 |
| Calculated nutrient composition^b^ |  |  |
| Digestible energy, MJ kg^-1^ | 13.14 | 13.14 |
| Crude protein, % | 15.20 | 15.20 |
| Lysine, % | 0.86 | 0.86 |
| Calcium, % | 0.86 | 0.86 |
| Total phosphorus, % | 0.69 | 0.69 |
| Available phosphorus, % | 0.41 | 0.41 |
| Folic acid, mg kg^-1^ | 1.30 | 16.30 |
| Choline, mg kg^-1^ | 1025.00 | 2230.00 |
| Vitamin B12, μg kg^-1^ | 30.00 | 150.00 |
| Vitamin B6, mg kg^-1^ | 3.00 | 1180.00 |
| Methionine, mg kg^-1^ | 2050.00 | 4700.00 |
| Nutrient composition, %^d^ |  |  |
| Crude protein | 15.26 | 15.22 |
| Crude ash | 5.96 | 5.92 |
| Crude fat | 5.63 | 5.57 |
| Crude fiber | 5.23 | 5.38 |
| Calcium | 0.94 | 0.98 |
| Total phosphorus | 0.64 | 0.64 |

^a^Provided the following per kg diet: 10,000 IU vitamin A; 1,000 IU vitamin D3; 60 IU vitamin E; 2 mg vitamin B1; 4 mg vitamin B2; 3mg niacin; 15 mg Cu; 110 mg Fe; 100 mg Zn; 20 mg Mn; 0.2 mg I; 0.3 mg Se.

^b^ Calculated values according to the China Feed Database.

^c^ The filler was wheat middlings (CF =2.8%, CP = 13.6%, DE = 12.98 MJ kg^-1^).

^d^The nutrient composition is measured value.

**Table S2.** Ingredients and nutrient composition of basal diets during lactation

| Items | Content |
| --- | --- |
| Ingredients % |  |
| Corn | 59.00 |
| Rice bran | 7.00 |
| Soybean meal | 22.00 |
| Fermented soybean meal | 4.00 |
| Soybean oil | 2.00 |
| Fish meal | 2.00 |
| Dicalcium phosphate | 1.40 |
| Limestone | 1.20 |
| NaCl | 0.30 |
| Lysine monohydrochloride, 98.5% | 0.05 |
| Choline chloride, 60% | 0.17 |
| Minerals^a^ | 0.20 |
| Vitamins^a^ | 0.20 |
| Filler^b^ | 0.48 |
| Total | 100.00 |
| Calculated nutrient composition^c^ |  |
| Digestible energy, MJ kg^-1^ | 13.81 |
| Crude protein, % | 18.46 |
| Lysine, % | 1.05 |
| Methionine, % | 0.30 |
| Threonine, % | 0.71 |
| Tryptophan, % | 0.22 |
| Calcium, % | 0.93 |
| Total phosphorus, % | 0.73 |
| Available phosphorus, % | 0.43 |
| Nutrient composition, %^d^ |  |
| Crude protein | 18.86 |
| Crude ash | 6.25 |
| Crude fat | 4.44 |
| Crude fiber | 4.05 |
| Calcium | 0.98 |
| Total phosphorus | 0.69 |

^a^Provided the following per kg diet:7000 IU vitamin A; 1500 IU vitamin D3; 16 IU vitamin E; 1 mg vitamin B1; 2.4 mg vitamin B2; 1mg vitamin B6; 8 μg vitamin B12; 20 mg niacin; 0.6 mg folic acid; 16 mg Cu; 100 mg Fe; 80 mg Zn; 2 mg Mn; 0.14 mg I; 0.1 mg Se.

^b^The filler was wheat middlings (CF =2.8%, CP = 13.6%, DE = 12.98 MJ kg^-1^).

^c^ Calculated values according to the China Feed Database.

^d^ The nutrient composition is measured value.


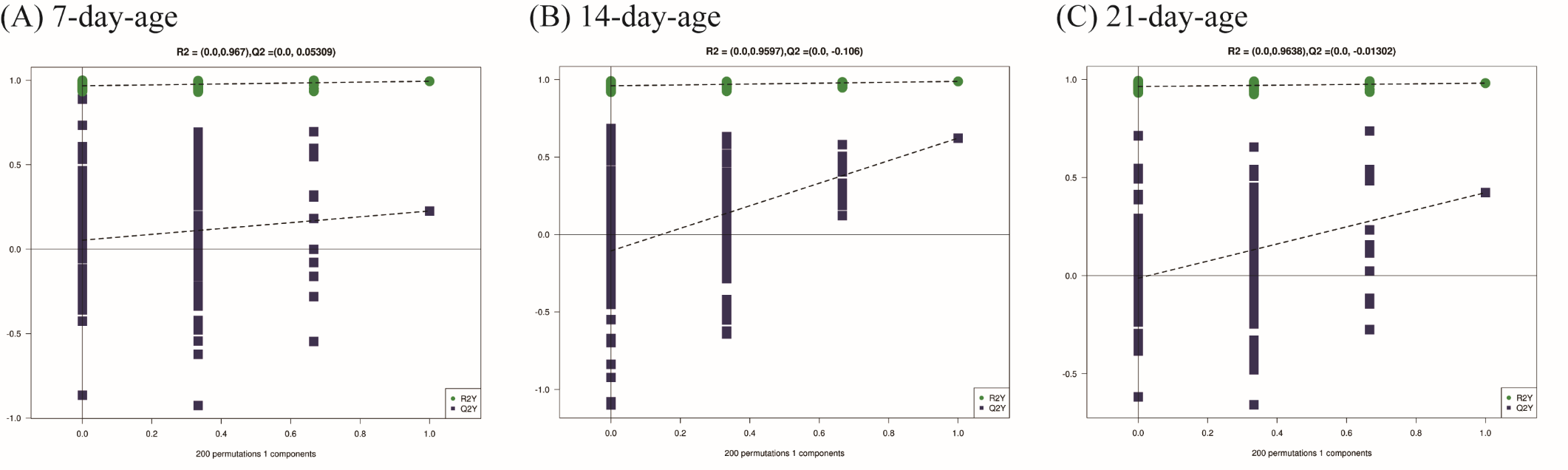


**Figure S1** The validation plots of the OPLS-DA analysis (orthogonal partial least squares discriminant analysis) of fecal metabolites in suckling piglets from sows fed MET vs. CON diet (n=6). Abbreviation: CON, the control group; MET, the methyl-donor micronutrients group.
